# Supplementary material for: Ostrich eggshell beads from Ga-Mohana Hill North Rockshelter, southern Kalahari, and the implications for understanding social networks during Marine Isotope Stage 2
Source: PLoS One. 2022 Jun 1;17(6):e0268943. doi: 10.1371/journal.pone.0268943 (PMC9159631; doi:10.1371/journal.pone.0268943)
Supplement: S1 Appendix — PDF document of RMarkdown version of the code for making the maps of southern African MIS 2 ostrich eggshell assemblages. (PDF) [file pone.0268943.s004.pdf]

Southern African Regional OES MIS2

Amy Hatton

29/06/2021

Ostrich eggshell beads from Ga-Mohana Hill North Rockshelter, southern Kalahari, and the implications for understanding social networks during Marine Isotope Stage 2

Authors: Amy Hatton, Benjamin Collins, Benjamin J. Schoville, Jayne Wilkins

Code for making the maps in this paper

Install Packages

Read in the csv and convert to a spatial object

```
sa_beads <- read.csv("../data/sa_oes_bead_table.csv")

#convert to sf (spatial) object
sa_beads_sf <- st_as_sf(sa_beads, coords = c("long","lat" ),
                        crs = 4326)
```

Get the distances between a few of the sites for the text

```
#distance between Dikbosch and GHN
st_distance(sa_beads_sf[23,], sa_beads_sf[25,])
```

```
## Units: [m]
##          [,1]
## [1,] 152774.4
```

```
#distance between BRS and HNK
st_distance(sa_beads_sf[12,], sa_beads_sf[29,])
```

```
## Units: [m]
##          [,1]
## [1,] 38935.69
```

Prep the Data for plotting the pie charts (preforms vs finished beads)

```
sa_beads <- clean_names(sa_beads)

sa_beads$beads_finished <- as.numeric(sa_beads$beads_finished)
sa_beads$beads_preforms <- as.numeric(sa_beads$beads_preforms)
sa_beads$oes_fragments <- as.numeric(sa_beads$oes_fragments)

beads_mis2 <- sa_beads %>%
  dplyr::select(site_abb, beads_finished,
                beads_preforms,oes_fragments,
                mean_diameter_mm, long, lat) %>%
  mutate(oes_total = beads_finished + beads_preforms + oes_fragments,
         bead_total = beads_finished + beads_preforms) %>%
  group_by(site_abb) %>%
  summarise(beads_finished = sum(beads_finished),
            beads_preforms = sum(beads_preforms),
            oes_fragments = sum(oes_fragments),
            bead_total = sum(bead_total),
            oes_total = sum(oes_total),
            diameter = mean(mean_diameter_mm),
            long = mean(long),
            lat = mean(lat)) %>%
  mutate(fin_prop = beads_finished/bead_total,
         pre_prop = beads_preforms/bead_total,
         fin_vs_oes_prop = beads_finished/oes_total,
         oes_prop = (beads_preforms + oes_fragments)/oes_total)
```

1) Plot a Raster of southern africa as base plot to plot the pie charts on top of.

Import the raster - this is a 90m resolution dem from Open Topograpgy that has been downsampled in R by a factor of 8. The raster file is too large to use otherwise and this resolution is fin enough for plotting.

```
#import raster
sa_dem <- raster("../data/sa_dem_downsampled.tif")
#import hillshade
hs <- raster("../data/sa_hs.tif")
```

2) Read in and process the ostrich species distribution data.

This is data from the South African Bird Atlas Project 2 (SABAP2) acessed at (http://sabap2.birdmap.africa/)

```
oes_dist <- st_read("../data/ostrich_distribution.gpkg")
```

```
## Reading layer `SABAP2_ostrich_distribution' from data source `C:\Users\Amy\Documents\
## Simple feature collection with 4771 features and 22 fields
## geometry type:  MULTIPOLYGON
## dimension:      XY
## bbox:           xmin: 12.16667 ymin: -34.8333 xmax: 39.16667 ymax: 0.8333333
## geographic CRS: WGS 84
```

Rasterise the OES data

```
ext <- extent(oes_dist)
r <- raster(ext, res=c(1/12,1/12))
#resolution of SABAP2 is 1/9
#(1/3 x 1/3) of a quarter degree lat long square
r <- rasterize(oes_dist, r, field="full.protocol")
```

Interpolate the OES distribution data using ordinary kriging

```
library(gstat)
library(automap)

#convert ostrich raster to points
p <- rasterToPoints(r, spatial = TRUE)

#interpolate
pred <- autoKrige(layer~1, p)
```

```
## [using ordinary kriging]
```

```
oes_pred <- pred$krige_output
```

Rasterise the interpolated oes data

```
#rasterise prediction
r1 <- raster(oes_pred)
#create raster with extent of the sa dem file
ext <- extent(sa_dem)
#ext <- extent(11.6,33, -34.2, -16.8)
r1 <- extend(r1, ext, value = NA)
```

Make the resolution of the oes distribution data finer to allow for plotting the coarser resolution makes a very pixelated map. This is alright because we just want a vague idea of the ostich distribution.

```
#make the resolution finer to allow for nice plotting
#(not good for analysis but fine for visualising)
r1_agg <- disaggregate(r1, fact=50)
```

```
#set the coordinate ref system
crs(r1_agg) <- "+proj=longlat +ellps=WGS84 +datum=WGS84 +no_defs"

#write the ostrich distribution raster
writeRaster(r1_agg, "../data/ostrich_distribution.tif", overwrite=TRUE)
```

Get the country borders for all southern african countries from Natural Earth

```
#get the polygons for other countries (zambia etc)
library(rnaturalearth)
s <- ne_countries(scale = "large", type = "countries",
                  continent = "africa",
                  returnclass =  "sf")
s1 <- s %>%
  filter(name == "Angola" | name == "Zambia" |
           name == "Zimbabwe"|name == "Mozambique" |
           name == "South Africa" | name == "eSwatini" |
           name == "Namibia" | name == "Botswana" |
           name == "Lesotho")
s1 <- st_transform(s1, 4326)

library(rgeos)
```

```
## rgeos version: 0.5-3, (SVN revision 634)
##  GEOS runtime version: 3.8.0-CAPI-1.13.1
##  Linking to sp version: 1.4-2
##  Polygon checking: TRUE
```

```
my_box <- rgeos::bbox2SP(n = -16.95106 , s = -34.82195 ,
                          w = 11.71762, e = 32.89308,
                          proj4string = CRS("+proj=longlat +ellps=WGS84
                                            +datum=WGS84 +no_defs"))
my_box <- st_as_sf(my_box, 4326)

#get the intersection of the sa bbox and this
a_borders <- st_intersection(my_box, s1)
```

```
## although coordinates are longitude/latitude, st_intersection assumes that they are pl
```

Crop the ostrich distribution data to the country borders

```
#crop to southern africa
r2 <- mask(r1_agg, a_borders)
oes_pred_sa <- crop(r2, a_borders)

#make stars object
```

```
oes_pred_stars <- st_as_stars(oes_pred_sa)
```

Convert the clipped dem to a df for plotting with ggplot

```
# convert to a df for plotting in two steps,
# First, to a SpatialPointsDataFrame
dem_pts <- rasterToPoints(sa_dem, spatial = TRUE)
# Then to a 'conventional' dataframe
dem_df  <- data.frame(dem_pts)
#rm(dem_pts, sa_dem)
```

Make the maps

Now that we've prepped all of the data layers for the maps we can make them

```
library(ggplot2) # For map design
library(ggspatial) # For map design
library(ggrepel)  # For map design
library(patchwork) # For multiple map layout
```

Base maps

```
##
## Attaching package: 'patchwork'

## The following object is masked from 'package:raster':
##
##     area
```

```
library(raster) # For manage raster data
library(sf) # For manage vector data
library(ggnewscale)

#set up a colour palette
pal <- rev(RColorBrewer::brewer.pal(8, "RdYlBu"))
pal1 <- rev(c("#d7191c", "#fdae61", "#ffffbf", "#abdda4", "#2b83ba"))
pal2 <- (RColorBrewer::brewer.pal(5, "BrBG"))
pal3 <- c("#defae1", "#addbb2", "#67b5a5", "#2986ab", "#2c5f96")

#convert to a stars object
hs_stars <- st_as_stars(hs)


#### Working plot
hillshade <- ggplot() +
  geom_stars(data =hs_stars, downsample = 5, show.legend = FALSE,
```

```
              alpha = 0.4)+
   scale_fill_continuous( low = "black", high = "white",
                          na.value = "white")+
   theme_bw() +
  coord_equal()+
    scale_x_continuous(expand=c(0,0))+
    scale_y_continuous(expand=c(0,0))


#layer 3 - ostrich distribution
p2 <- hillshade +
  new_scale("fill")+
  geom_stars(data = oes_pred_stars, alpha = 0.6) +
  scale_fill_gradientn(colours = pal3, limits = c(0, 100),
                       na.value = "white") +
  labs(x = "Longitude", y = "Latitude",
       fill = "Modern Prevalence \nof Ostriches") +
  theme_bw()

# layer 4 country borders
#add the country borders
p3 <- p2 +
    geom_sf(data = a_borders, size =1.2, colour = "white", fill = NA)
```

```
## Coordinate system already present. Adding new coordinate system, which will replace t
```

```
#add the pie charts at sites for proportion beads to preforms

library(scatterpie)
```

Map with sites and pie charts for preform/bead ratio

```
##
## Attaching package: 'scatterpie'
```

```
## The following object is masked from 'package:sp':
##
##     recenter
```

```
library(ggrepel)

#mutate the lat and long column names for geom_scatterpie to work
beads_mis2 <- beads_mis2 %>%
  mutate(long_geom = long) %>%
  mutate(lat_geom = lat)
```

```r
bead_data <- beads_mis2
pre_vs_oes <- beads_mis2 %>%
  filter_at(vars(pre_prop, fin_prop), any_vars(!is.na(.)))

map1 <- p3+
  new_scale("fill")+
  geom_scatterpie(aes(x=long_geom, y=lat_geom, r=0.6),
                  data=pre_vs_oes, cols=c("fin_prop", "pre_prop"),
                  alpha = 0.8)+
  scale_fill_manual(name = "Type",
                    labels = c("Finished Beads", "Preforms"),
                    values = c("#fc9595", "#b488c8"))+
  geom_text_repel(data = pre_vs_oes, aes(x=long_geom,
                                        y=lat_geom, label=site_abb),
                  point.padding =2, box.padding = 0.5)+
  theme(legend.position = "bottom")


pdf("../plots/fig3.pdf")
print(map1)
dev.off()
```

```
## pdf
##   2
```

```r

#filter out na's
bead_vs_oes <- beads_mis2 %>%
  filter_at(vars(fin_vs_oes_prop, oes_prop), any_vars(!is.na(.)))

map2 <- p3+
  new_scale("fill")+
  geom_scatterpie(aes(x=long_geom, y=lat_geom, r=0.6),
                  data=bead_vs_oes,
                  cols=c("fin_vs_oes_prop", "oes_prop"), alpha = 0.8)+

  scale_fill_manual(name = "Type", labels = c("Finished Beads",
                                              "Preforms and\nOES Fragments"),
                    values = c("#fc9595", "#b488c8"))+
  geom_text_repel(data = bead_vs_oes, aes(x=long_geom, y=lat_geom,
                                         label=site_abb), point.padding =2,
                  box.padding = 0.5)+
  theme(legend.position = "bottom")
```

```r
pdf("../plots/fig4.pdf")
print(map2)
dev.off()
```

Map with frequencies of bead vs preforms +frags

```
## pdf
##   2
```

```r
library(viridis)
```

Map with OES bead + preform abundance

```
## Loading required package: viridisLite
```

```r
bead_count <- beads_mis2 %>%
  filter_at(vars(bead_total), any_vars(!is.na(.)))

range(bead_count$bead_total)
```

```
## [1]   0 184
```

```r
map3 <- p3 +
  new_scale("fill")+
  geom_point( data=bead_count, aes(x=long_geom, y=lat_geom,
                                   size=bead_total, fill=bead_total),
              shape = 21, alpha = 0.7) +
  scale_size_continuous(range=c(1,10)) +
  scale_fill_viridis( option = "plasma", guide = "legend") +
  geom_text_repel(data = bead_count, aes(x=long_geom, y=lat_geom,
                                         label=site_abb), point.padding =2,
                  box.padding = 0.5)+
  theme(legend.position = "bottom")+
  guides(fill=guide_legend("OES bead and preform\nabundance"),
         size=guide_legend("OES bead and preform\nabundance"))

pdf("../plots/fig5.pdf")
print(map3)
dev.off()
```

```
## pdf
##   2
```

```r
oes_count <- beads_mis2 %>%
  filter_at(vars(oes_total), any_vars(!is.na(.)))
```

```
range(oes_count$oes_total)
```

Map with OES abundance (circles with diameter showing number of oes)

```
## [1]     1 12273
```

```
map4 <- p3 +
  new_scale("fill")+
  geom_point( data=oes_count, aes(x=long_geom, y=lat_geom,
                                  size=oes_total, fill=oes_total),
              shape = 21, alpha = 0.7) +
  scale_size_continuous(range=c(1,10)) +
  scale_fill_viridis( option = "plasma", guide = "legend") +
  geom_text_repel(data = oes_count, aes(x=long_geom,
                                        y=lat_geom, label=site_abb),
                  point.padding =2, box.padding = 0.5)+
  theme(legend.position = "bottom")+
  guides(fill=guide_legend("OES Fragment\nAbundance"),
         size=guide_legend("OES Fragment\nAbundance"))

pdf("../plots/fig6.pdf")
print(map4)
dev.off()
```

```
## pdf
##   2
```

```
bead_diam <- bead_data %>%
  filter_at(vars(diameter), any_vars(!is.na(.)))

map5 <- p3 +
  new_scale("fill")+
  geom_point( data=bead_diam, aes(x=long_geom, y=lat_geom,
                                  size=diameter, fill=diameter),
              shape = 21, alpha = 0.7) +
  scale_size_continuous(range=c(2,7)) +
  scale_fill_viridis( option = "plasma", guide = "legend") +
  geom_text_repel(data = bead_diam, aes(x=long_geom, y=lat_geom,
                                        label=site_abb), point.padding =2,
                  box.padding = 0.5)+
  theme(legend.position = "bottom")+
  guides(fill=guide_legend("Bead diameter (mm)"),
         size=guide_legend("Bead diameter (mm)"))

pdf("../plots/fig7.pdf")
```

```
print(map5)
dev.off()
```

Map with diameter of beads

```
## pdf
##   2
```
