# Supplementary material for: Ostrich eggshell beads from Ga-Mohana Hill North Rockshelter, southern Kalahari, and the implications for understanding social networks during Marine Isotope Stage 2
Source: PLoS One. 2022 Jun 1;17(6):e0268943. doi: 10.1371/journal.pone.0268943 (PMC9159631; doi:10.1371/journal.pone.0268943)
Supplement: S2 Appendix — PDF document of RMarkdown version of the code for statistical analysis of southern African MIS 2 ostrich eggshell assemblages and Ga-Mohana Hill North Rockshelter assemblages. (PDF) [file pone.0268943.s005.pdf]

Statistical Analysis OES Paper

Amy Hatton

29/06/2021

Ostrich eggshell beads from Ga-Mohana Hill North Rockshelter, southern Kalahari, and the implications for understanding social networks during Marine Isotope Stage 2

Authors: Amy Hatton, Benjamin Collins, Benjamin J. Schoville, Jayne Wilkins

Code for statistical analysis in this paper

Chi-squared test to compare levels of burning between OES frags and beads

Read in OES data and process

```
oesbead_data <- read.csv("../data/ghn_oesbeads.csv")
oes_data <- read.csv("../data/ghn_oes.csv")
```

Filter out the DBGS points

```
# filter out only the DBGS finds
oes_beads_dbgs <- oesbead_data %>%
  filter(Strat.Agg == "DBGS") %>%
  clean_names() %>%
  mutate(colour = color)
oes_dbgs <- oes_data %>%
  filter(Strat.Agg == "DBGS")%>%
  clean_names()
```

Get the range and sd of some of the technological variables

```
range(oes_dbgs$length_mm)
```

```
## [1]  9.69 21.95
```

```r
sd(oes_dbgs$length_mm)
```

```
## [1] 2.890703
```

```r
range(oes_dbgs$width_mm)
```

```
## [1]  4.58 14.73
```

```r
sd(oes_dbgs$width_mm)
```

```
## [1] 2.559344
```

```r
range(oes_dbgs$weight_g)
```

```
## [1] 0.12 0.84
```

```r
sd(oes_dbgs$weight_g)
```

```
## [1] 0.1648607
```

```r
diam_1 <- oes_beads_dbgs %>%
  filter(bead_or_preform == "bead")
sd(diam_1$max_ext_di)
```

```
## [1] 0.4261352
```

```r
sd(diam_1$max_aper_di)
```

```
## [1] 0.2479483
```

```r
sd(oes_beads_dbgs$max_thick)
```

```
## [1] 0.1851594
```

Combine OES frags and OES beads as one df

```r
oes_beads_dbgs <- oes_beads_dbgs %>%
  mutate(type = "bead_or_preform") %>%
  dplyr::select(lot, find, type, colour)

oes_dbgs <- oes_dbgs %>%
  mutate(type = "fragment")%>%
  dplyr::select(lot, find, type, colour)

#combine the two into 1 df
dbgs_oes <- rbind(oes_beads_dbgs,oes_dbgs)
```

Check that the levels for colour are clean

```r
dbgs_oes$colour <- as.factor(dbgs_oes$colour)
levels(dbgs_oes$colour)
```

```
## [1] "black"   "brown"   "red"     "unburnt" "yellow"
```

```
#need to subsume brown into unburnt (brown is just staining from sediment)
levels(dbgs_oes$colour) <- c("black"  , "unburnt"  , "red"   ,  "unburnt" ,"yellow" )
```

Chi-squared test

Create contigency table

```
library(MASS)       # load the MASS package
```

```
##
## Attaching package: 'MASS'

## The following object is masked from 'package:dplyr':
##
##     select
```

```
tbl <- table(dbgs_oes$colour, dbgs_oes$type)
```

Run chi-sqaured test

```
chisq.test(tbl)
```

```
## Warning in stats::chisq.test(x, y, ...): Chi-squared approximation may be
## incorrect

##
##  Pearson's Chi-squared test
##
## data:  tbl
## X-squared = 8.5855, df = 3, p-value = 0.03534
```

Anova for oes bead mean diameter

Read in the data

```
sa_beads <- read.csv("../data/sa_oes_bead_table.csv")

sa_beads <- clean_names(sa_beads)
```

Process the data

```
#group by site
bead_diam <- sa_beads %>%
  dplyr::select(site, site_abb,mean_diameter_mm) %>%
  filter(!is.na(mean_diameter_mm)) %>%
  group_by(site) %>%
  mutate(diameter = mean(mean_diameter_mm))
```

```r
bead_diam_mean <- sa_beads %>%
  dplyr::select(site, site_abb,mean_diameter_mm) %>%
  filter(!is.na(mean_diameter_mm)) %>%
  group_by(site) %>%
  mutate(diameter = mean(mean_diameter_mm)) %>%
  dplyr::select(!mean_diameter_mm) %>%
  distinct()
```

Conduct anova test

```r
# Compute the analysis of variance
res.aov <- aov(mean_diameter_mm ~ site, data = bead_diam)
# Summary of the analysis
summary(res.aov)
```

```
##             Df Sum Sq Mean Sq F value Pr(>F)
## site         5  5.638  1.1276   9.935 0.0124 *
## Residuals    5  0.567  0.1135
## ---
## Signif. codes:  0 '***' 0.001 '**' 0.01 '*' 0.05 '.' 0.1 ' ' 1
```

```r
#plot(res.aov)

#Tukey pairwise comparisons
TukeyHSD(res.aov)
```

```
##   Tukey multiple comparisons of means
##     95% family-wise confidence level
##
## Fit: aov(formula = mean_diameter_mm ~ site, data = bead_diam)
##
## $site
##                                                            diff        lwr
## Bushman's Rock Shelter-Buffelskloof                       1.000 -0.7601711
## Dikbosch 1-Buffelskloof                                   0.175 -1.4318090
## Ga-Mohana Hill  North Rockshelter-Buffelskloof            0.100 -1.9324705
## Grassridge-Buffelskloof                                  -0.800 -2.8324705
## Ha Makotoko-Buffelskloof                                 -1.200 -2.9601711
## Dikbosch 1-Bushman's Rock Shelter                        -0.825 -2.0696289
## Ga-Mohana Hill  North Rockshelter-Bushman's Rock Shelter -0.900 -2.6601711
## Grassridge-Bushman's Rock Shelter                        -1.800 -3.5601711
## Ha Makotoko-Bushman's Rock Shelter                       -2.200 -3.6371737
## Ga-Mohana Hill  North Rockshelter-Dikbosch 1             -0.075 -1.6818090
## Grassridge-Dikbosch 1                                    -0.975 -2.5818090
## Ha Makotoko-Dikbosch 1                                   -1.375 -2.6196289
## Grassridge-Ga-Mohana Hill  North Rockshelter             -0.900 -2.9324705
```

```
## Ha Makotoko-Ga-Mohana Hill  North Rockshelter             -1.300 -3.0601711
## Ha Makotoko-Grassridge                                    -0.400 -2.1601711
##                                                                   upr     p adj
## Bushman's Rock Shelter-Buffelskloof                        2.76017108 0.2973850
## Dikbosch 1-Buffelskloof                                    1.78180901 0.9956014
## Ga-Mohana Hill  North Rockshelter-Buffelskloof             2.13247050 0.9999007
## Grassridge-Buffelskloof                                    1.23247050 0.5921345
## Ha Makotoko-Buffelskloof                                   0.56017108 0.1830156
## Dikbosch 1-Bushman's Rock Shelter                          0.41962891 0.1984408
## Ga-Mohana Hill  North Rockshelter-Bushman's Rock Shelter   0.86017108 0.3769759
## Grassridge-Bushman's Rock Shelter                         -0.03982892 0.0458952
## Ha Makotoko-Bushman's Rock Shelter                        -0.76282633 0.0085525
## Ga-Mohana Hill  North Rockshelter-Dikbosch 1               1.53180901 0.9999234
## Grassridge-Dikbosch 1                                      0.63180901 0.2522778
## Ha Makotoko-Dikbosch 1                                    -0.13037109 0.0339086
## Grassridge-Ga-Mohana Hill  North Rockshelter               1.13247050 0.4947924
## Ha Makotoko-Ga-Mohana Hill  North Rockshelter              0.46017108 0.1436932
## Ha Makotoko-Grassridge                                     1.36017108 0.9101716
```

Check which sites have more than 50 beads

```
ba <- sa_beads %>%
  mutate(bead_total = beads_finished + beads_preforms ) %>%
  filter(bead_total > 50)

range(ba$bead_total)
```

```
## [1]  74 170
```

Spearman rank order correlation

Run Spearman rank order correlation on ostrich prevalence and bead size

```
library(raster)
```

```
## Loading required package: sp

##
## Attaching package: 'raster'

## The following objects are masked from 'package:MASS':
##
##     area, select

## The following object is masked from 'package:janitor':
##
##     crosstab

## The following object is masked from 'package:dplyr':
```

```
##
##     select
```

```r
library(sf)
```

```
## Linking to GEOS 3.8.0, GDAL 3.0.4, PROJ 6.3.1
```

```r
# read in oes distribution raster
oes_dist <- raster("../data/ostrich_distribution.tif")

sa_beads_sf <- st_as_sf(sa_beads, coords = c("long","lat" ),
                     crs = 4326)


bead_diam_sf <-  sa_beads_sf %>%
  dplyr::select(site, site_abb,mean_diameter_mm) %>%
  filter(!is.na(mean_diameter_mm)) %>%
  group_by(site) %>%
  mutate(diameter = mean(mean_diameter_mm)) %>%
  dplyr::select(!mean_diameter_mm) %>%
  distinct(.keep_all=TRUE)

#get the raster values (ostrich prevalence) for a 5km area around each site

ras_value <- raster::extract(oes_dist, bead_diam_sf, buffer= 5000, fun=mean)

ras_value1 <- raster::extract(oes_dist, sa_beads_sf, buffer= 5000, fun=mean)

val_points <- cbind(bead_diam_sf, ras_value)
```
